# 3,5-Dimeth­oxy-2-[(4-propyl­phen­yl)imino­meth­yl]phenol

**DOI:** 10.1107/S1600536809007958

**Published:** 2009-03-11

**Authors:** Zarife Sibel Şahin, Ayşen Alaman Ağar, Ferda Erşahin, Şamil Işık

**Affiliations:** a Department of Physics, Faculty of Arts and Sciences, Ondokuz Mayıs University, Kurupelit, TR-55139 Samsun, Turkey; b Department of Chemistry, Arts and Sciences Faculty, Ondokuz Mayıs University, 55139 Samsun, Turkey

## Abstract

The title compound, C_18_H_21_NO_3_, crystallizes in the phenol–imine tautomeric form, with the H atom attached to oxygen rather than on nitro­gen. This H atom is involved in a strong intra­molecular O—H⋯N hydrogen bond. A C—H⋯π inter­action is also present. The dihedral angle between the aromatic rings is 12.23 (7)°.

## Related literature

Schiff base compounds can be classified by their photochromic and thermochromic characteristics, see: Cohen *et al.* (1964 ▶); Hadjoudis *et al.* (1987 ▶); Calligaris *et al.* (1972 ▶); Hökelek *et al.* (2000 ▶); Dey *et al.* (2001 ▶); Ünver *et al.* (2002 ▶); Karadayı *et al.* (2003 ▶). Bernstein *et al.* (1995 ▶) describe the use of graph-set models for the description of hydrogen bonds.
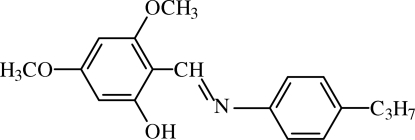


## Experimental

### 

#### Crystal data

C_18_H_21_NO_3_
*M* *_r_* = 299.36
Monoclinic, 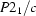

*a* = 15.1143 (15) Å
*b* = 7.2587 (5) Å
*c* = 17.737 (2) Å
β = 123.669 (7)°
*V* = 1619.5 (3) Å^3^
*Z* = 4
Mo *K*α radiation
μ = 0.08 mm^−1^
*T* = 296 K
0.48 × 0.26 × 0.12 mm

#### Data collection

Stoe IPDS-II diffractometer
Absorption correction: none
18964 measured reflections
3353 independent reflections
1337 reflections with *I* > 2σ(*I*)
*R*_int_ = 0.075

#### Refinement

*R*[*F* ^2^ > 2σ(*F* ^2^)] = 0.052
*wR*(*F* ^2^) = 0.145
*S* = 0.86
3353 reflections
200 parameters
H-atom parameters constrained
Δρ_max_ = 0.14 e Å^−3^
Δρ_min_ = −0.21 e Å^−3^

### 

Data collection: *X-AREA* (Stoe & Cie, 2002 ▶); cell refinement: *X-AREA*; data reduction: *X-RED32* (Stoe & Cie, 2002 ▶); program(s) used to solve structure: *SHELXS97* (Sheldrick, 2008 ▶); program(s) used to refine structure: *SHELXL97* (Sheldrick, 2008 ▶); molecular graphics: *ORTEP-3 for Windows* (Farrugia, 1997 ▶); software used to prepare material for publication: *WinGX* (Farrugia, 1999 ▶).

## Supplementary Material

Crystal structure: contains datablocks I, global. DOI: 10.1107/S1600536809007958/zl2174sup1.cif

Structure factors: contains datablocks I. DOI: 10.1107/S1600536809007958/zl2174Isup2.hkl

Additional supplementary materials:  crystallographic information; 3D view; checkCIF report

## Figures and Tables

**Table 1:** Hydrogen-bond geometry (Å, °)

*D*—H⋯*A*	*D*—H	H⋯*A*	*D*⋯*A*	*D*—H⋯*A*
O2—H2⋯N1	0.82	1.87	2.602 (2)	148
C18—H18*B*⋯*Cg*2^i^	0.96	2.80	3.764 (3)	178

Symmetry code: (i) 
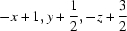
. *Cg*2 is the centroid of the C7–C12 ring.

**Fig. 1.**
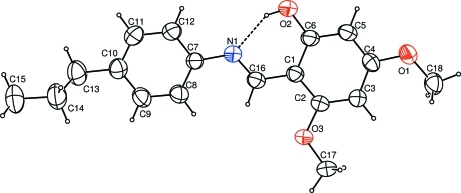
The molecular structure of the title compound, showing the atom-numbering scheme. Displacement ellipsoids are drawn at the 30% probability. The dashed line represents the O-H···N hydrogen bond.

**Fig. 2.**
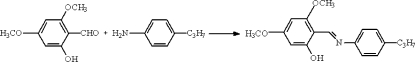
Synthesis of the title compound.

## supplementary crystallographic information

### Comment

Schiff bases have been extensively used as ligands in the field of coordination chemistry (Calligaris *et al.*, 1972). There are two characteristic properties of Schiff bases, *viz*. photochromism and thermochromism (Cohen *et al.*, 1964). These properties result from proton transfer from the hydroxyl O atom to the imine N atom (Hadjoudis *et al.*, 1987). Schiff bases display two possible tautomeric forms, namely the phenol-imine (Dey *et al.*, 2001; Karadayı *et al.*, 2003) and keto-amine (Hökelek *et al.*, 2000; Ünver *et al.*, 2002) forms.

In the structure of the title compound the N1—C16 bond length of 1.280 (3)Å is typical of a double bond. The dihedral angle between the C1—C6 and C8—C13 benzene rings is 12.23 (7)°. The C1—C16—N1—C7 torsion angle is 178.7 (2)°. Fig. 1 also shows a strong intramolecular hydrogen bond (O2—H2···N1) which can be described with an S(6) graph set motif (Bernstein *et al.*,1995). The compound also contains one intermolecular C—H···π interaction. Atom C18 in the molecule at (*x*, *y*, *z*) acts as hydrogen-bond donor to the centroid *Cg*2 of the ring C7—C12 in the molecule at (1 - *x*, 1/2 + *y*, 3/2 - *z*), thus forming a chain running parallel to the [010] direction. The details of C—H···π interaction are given in Table 1.

### Experimental

The compound 3,5-dimethoxy-2-[(4-propylphenylimino)methyl]phenol was prepared by refluxing of a mixture of a solution containing 2-hydroxy-4,6-dimethoxy-benzaldehyde (0.0236 g 0.129 mmol) in 20 ml ethanol and a solution containing 4-propylaniline (0.0175 g 0.129 mmol) in 20 ml ethanol. The reaction mixture was stirred for 1 h under reflux. Recrystallization from ethanol gave the pure product. The crystals of 4,6-dimethoxy-2-[(4-propylphenylimino)methyl]phenol suitable for X-ray analysis were obtained from ethylalcohol by slow evaporation (yield % 67; m.p.346–348 K) (Fig. 2).

### Refinement

The O—H hydrogen bond was placed in a calculated position with an O—H distance of 0.82Å, but was alloed to rotate around the C—O bond at a fixed angle to best fit the experimental electron density. The H was refined *U*_iso_(H) = 1.5*U*_eq_(O). The other H atoms attached to C atoms were refined using a riding model with C—H = 0.93Å and *U*_iso_(H) = 1.2*U*_eq_(C) for aromatic C atoms, C—H = 0.97Å and *U*_iso_(H) = 1.2*U*_eq_(C) for methylene C atoms and C—H = 0.96Å and *U*_iso_(H) = 1.5*U*_eq_(C) for methyl C atoms.

### Crystal data

**Table:**

C_18_H_21_NO_3_	*F*(000) = 640
*M**_r_* = 299.36	*D*_x_ = 1.228 Mg m^−^^3^
Monoclinic, *P*2_1_/*c*	Mo *K*α radiation, λ = 0.71073 Å
Hall symbol: -P 2ybc	Cell parameters from 10898 reflections
*a* = 15.1143 (15) Å	θ = 1.4–28.0°
*b* = 7.2587 (5) Å	µ = 0.08 mm^−^^1^
*c* = 17.737 (2) Å	*T* = 296 K
β = 123.669 (7)°	Prism, yellow
*V* = 1619.5 (3) Å^3^	0.48 × 0.26 × 0.12 mm
*Z* = 4	

### Data collection

**Table:**

Stoe IPDS-II diffractometer	1337 reflections with *I* > 2σ(*I*)
Radiation source: fine-focus sealed tube	*R*_int_ = 0.075
graphite	θ_max_ = 26.5°, θ_min_ = 1.6°
Detector resolution: 6.67 pixels mm^-1^	*h* = −18→18
ω scans	*k* = −8→9
18964 measured reflections	*l* = −22→22
3353 independent reflections	

### Refinement

**Table:**

Refinement on *F*^2^	Primary atom site location: structure-invariant direct methods
Least-squares matrix: full	Secondary atom site location: difference Fourier map
*R*[*F*^2^ > 2σ(*F*^2^)] = 0.052	Hydrogen site location: inferred from neighbouring sites
*wR*(*F*^2^) = 0.145	H-atom parameters constrained
*S* = 0.86	*w* = 1/[σ^2^(*F*_o_^2^) + (0.0666*P*)^2^] where *P* = (*F*_o_^2^ + 2*F*_c_^2^)/3
3353 reflections	(Δ/σ)_max_ < 0.001
200 parameters	Δρ_max_ = 0.14 e Å^−^^3^
0 restraints	Δρ_min_ = −0.21 e Å^−^^3^

### Special details

**Table:**

Geometry. All e.s.d.'s (except the e.s.d. in the dihedral angle between two l.s. planes) are estimated using the full covariance matrix. The cell e.s.d.'s are taken into account individually in the estimation of e.s.d.'s in distances, angles and torsion angles; correlations between e.s.d.'s in cell parameters are only used when they are defined by crystal symmetry. An approximate (isotropic) treatment of cell e.s.d.'s is used for estimating e.s.d.'s involving l.s. planes.
Refinement. Refinement of *F*^2^ against ALL reflections. The weighted *R*-factor *wR* and goodness of fit *S* are based on *F*^2^, conventional *R*-factors *R* are based on *F*, with *F* set to zero for negative *F*^2^. The threshold expression of *F*^2^ > σ(*F*^2^) is used only for calculating *R*-factors(gt) *etc*. and is not relevant to the choice of reflections for refinement. *R*-factors based on *F*^2^ are statistically about twice as large as those based on *F*, and *R*- factors based on ALL data will be even larger.

### Fractional atomic coordinates and isotropic or equivalent isotropic displacement parameters (Å^2^)

**Table:**

	*x*	*y*	*z*	*U*_iso_*/*U*_eq_	
C1	0.53442 (17)	0.1004 (3)	0.80353 (15)	0.0667 (6)	
C2	0.57933 (17)	0.1123 (3)	0.75252 (15)	0.0672 (6)	
C3	0.68738 (17)	0.1079 (3)	0.79200 (16)	0.0722 (7)	
H3	0.7155	0.1170	0.7569	0.087*	
C4	0.75352 (18)	0.0896 (3)	0.88549 (17)	0.0730 (7)	
C5	0.71280 (19)	0.0762 (4)	0.93799 (17)	0.0825 (8)	
H5	0.7584	0.0629	1.0004	0.099*	
C6	0.60502 (18)	0.0825 (3)	0.89829 (16)	0.0732 (7)	
C7	0.26549 (18)	0.0884 (3)	0.76044 (16)	0.0678 (6)	
C8	0.1924 (2)	0.1356 (4)	0.67118 (18)	0.0873 (8)	
H8	0.2159	0.1722	0.6348	0.105*	
C9	0.0850 (2)	0.1289 (4)	0.63550 (19)	0.0919 (8)	
H9	0.0375	0.1597	0.5749	0.110*	
C10	0.0452 (2)	0.0787 (4)	0.6858 (2)	0.0817 (7)	
C11	0.1189 (2)	0.0343 (4)	0.7747 (2)	0.0931 (9)	
H11	0.0953	0.0002	0.8112	0.112*	
C12	0.2272 (2)	0.0384 (4)	0.81199 (18)	0.0870 (8)	
H12	0.2745	0.0069	0.8725	0.104*	
C16	0.42105 (17)	0.1008 (3)	0.76044 (16)	0.0719 (7)	
H16	0.3775	0.1081	0.6976	0.086*	
C18	0.90908 (19)	0.1115 (4)	0.8815 (2)	0.1004 (9)	
H18A	0.9850	0.1025	0.9219	0.151*	
H18B	0.8904	0.2318	0.8546	0.151*	
H18C	0.8840	0.0200	0.8349	0.151*	
C17	0.5443 (2)	0.1229 (5)	0.60388 (16)	0.0988 (9)	
H17A	0.4853	0.1357	0.5421	0.148*	
H17B	0.5792	0.0074	0.6113	0.148*	
H17C	0.5936	0.2219	0.6191	0.148*	
C13	−0.0731 (2)	0.0740 (5)	0.6453 (2)	0.1140 (11)	
H13A	−0.0832	0.0528	0.6941	0.137*	
H13B	−0.1018	0.1950	0.6205	0.137*	
C14	−0.1353 (2)	−0.0597 (5)	0.5758 (2)	0.1255 (12)	
H14A	−0.1062	−0.1811	0.5996	0.151*	
H14B	−0.1278	−0.0367	0.5257	0.151*	
C15	−0.2534 (2)	−0.0615 (5)	0.5394 (2)	0.1338 (14)	
H15A	−0.2886	−0.1549	0.4937	0.201*	
H15B	−0.2838	0.0566	0.5136	0.201*	
H15C	−0.2621	−0.0872	0.5880	0.201*	
N1	0.37759 (14)	0.0914 (3)	0.80506 (13)	0.0738 (6)	
O1	0.86157 (12)	0.0821 (3)	0.93083 (11)	0.0921 (6)	
O2	0.56789 (13)	0.0725 (3)	0.95188 (11)	0.0995 (7)	
H2	0.5027	0.0758	0.9205	0.149*	
O3	0.50721 (11)	0.1284 (3)	0.66174 (10)	0.0869 (6)	

### Atomic displacement parameters (Å^2^)

**Table:**

	*U*^11^	*U*^22^	*U*^33^	*U*^12^	*U*^13^	*U*^23^
C1	0.0666 (14)	0.0706 (17)	0.0586 (14)	−0.0048 (12)	0.0320 (13)	−0.0038 (13)
C2	0.0695 (15)	0.0707 (17)	0.0538 (14)	−0.0022 (12)	0.0293 (13)	−0.0031 (13)
C3	0.0699 (15)	0.0791 (18)	0.0677 (16)	−0.0023 (13)	0.0382 (13)	−0.0020 (15)
C4	0.0604 (14)	0.0728 (18)	0.0689 (16)	−0.0079 (12)	0.0254 (13)	−0.0030 (14)
C5	0.0741 (16)	0.106 (2)	0.0550 (14)	−0.0098 (15)	0.0280 (13)	−0.0006 (15)
C6	0.0744 (16)	0.0859 (19)	0.0553 (15)	−0.0108 (13)	0.0334 (14)	−0.0040 (14)
C7	0.0701 (15)	0.0712 (17)	0.0626 (15)	0.0008 (13)	0.0370 (14)	0.0005 (13)
C8	0.0779 (17)	0.100 (2)	0.0752 (18)	−0.0001 (15)	0.0368 (15)	0.0181 (16)
C9	0.0726 (17)	0.103 (2)	0.0811 (19)	0.0046 (15)	0.0309 (15)	0.0133 (17)
C10	0.0770 (16)	0.0770 (19)	0.094 (2)	0.0042 (14)	0.0491 (17)	−0.0086 (16)
C11	0.092 (2)	0.112 (2)	0.094 (2)	0.0066 (17)	0.0632 (19)	−0.0021 (19)
C12	0.0814 (18)	0.111 (2)	0.0739 (17)	0.0118 (15)	0.0463 (16)	0.0044 (15)
C16	0.0728 (15)	0.0767 (18)	0.0590 (14)	0.0011 (13)	0.0322 (13)	−0.0028 (14)
C18	0.0740 (16)	0.115 (2)	0.109 (2)	−0.0058 (16)	0.0486 (17)	0.0204 (19)
C17	0.0904 (17)	0.151 (3)	0.0602 (16)	0.0001 (18)	0.0451 (15)	−0.0106 (18)
C13	0.0812 (18)	0.124 (3)	0.131 (3)	0.0047 (18)	0.0549 (19)	−0.025 (2)
C14	0.0813 (19)	0.153 (3)	0.137 (3)	−0.007 (2)	0.057 (2)	−0.023 (3)
C15	0.0733 (19)	0.149 (3)	0.162 (3)	−0.0030 (19)	0.054 (2)	0.006 (3)
N1	0.0709 (12)	0.0871 (16)	0.0628 (12)	−0.0019 (11)	0.0367 (11)	−0.0005 (11)
O1	0.0646 (10)	0.1177 (16)	0.0783 (12)	−0.0085 (10)	0.0298 (10)	0.0050 (11)
O2	0.0793 (11)	0.156 (2)	0.0590 (10)	−0.0110 (13)	0.0359 (9)	0.0003 (12)
O3	0.0715 (10)	0.1344 (16)	0.0519 (10)	0.0047 (10)	0.0324 (9)	0.0013 (11)

### Geometric parameters (Å, °)

**Table:**

C1—C2	1.403 (3)	C11—H11	0.9300
C1—C6	1.411 (3)	C12—H12	0.9300
C1—C16	1.438 (3)	C16—N1	1.280 (3)
C2—O3	1.360 (2)	C16—H16	0.9300
C2—C3	1.375 (3)	C18—O1	1.422 (3)
C3—C4	1.389 (3)	C18—H18A	0.9600
C3—H3	0.9300	C18—H18B	0.9600
C4—O1	1.364 (3)	C18—H18C	0.9600
C4—C5	1.375 (3)	C17—O3	1.417 (2)
C5—C6	1.371 (3)	C17—H17A	0.9600
C5—H5	0.9300	C17—H17B	0.9600
C6—O2	1.347 (3)	C17—H17C	0.9600
C7—C12	1.374 (3)	C13—C14	1.435 (4)
C7—C8	1.380 (3)	C13—H13A	0.9700
C7—N1	1.418 (3)	C13—H13B	0.9700
C8—C9	1.379 (3)	C14—C15	1.525 (3)
C8—H8	0.9300	C14—H14A	0.9700
C9—C10	1.373 (3)	C14—H14B	0.9700
C9—H9	0.9300	C15—H15A	0.9600
C10—C11	1.373 (4)	C15—H15B	0.9600
C10—C13	1.514 (3)	C15—H15C	0.9600
C11—C12	1.385 (3)	O2—H2	0.8200
			
C2—C1—C6	117.2 (2)	N1—C16—H16	118.7
C2—C1—C16	121.1 (2)	C1—C16—H16	118.7
C6—C1—C16	121.6 (2)	O1—C18—H18A	109.5
O3—C2—C3	123.5 (2)	O1—C18—H18B	109.5
O3—C2—C1	114.34 (19)	H18A—C18—H18B	109.5
C3—C2—C1	122.2 (2)	O1—C18—H18C	109.5
C2—C3—C4	118.4 (2)	H18A—C18—H18C	109.5
C2—C3—H3	120.8	H18B—C18—H18C	109.5
C4—C3—H3	120.8	O3—C17—H17A	109.5
O1—C4—C5	115.9 (2)	O3—C17—H17B	109.5
O1—C4—C3	122.8 (2)	H17A—C17—H17B	109.5
C5—C4—C3	121.3 (2)	O3—C17—H17C	109.5
C6—C5—C4	120.0 (2)	H17A—C17—H17C	109.5
C6—C5—H5	120.0	H17B—C17—H17C	109.5
C4—C5—H5	120.0	C14—C13—C10	117.5 (3)
O2—C6—C5	118.5 (2)	C14—C13—H13A	107.9
O2—C6—C1	120.6 (2)	C10—C13—H13A	107.9
C5—C6—C1	120.9 (2)	C14—C13—H13B	107.9
C12—C7—C8	117.7 (2)	C10—C13—H13B	107.9
C12—C7—N1	116.5 (2)	H13A—C13—H13B	107.2
C8—C7—N1	125.8 (2)	C13—C14—C15	115.1 (3)
C9—C8—C7	120.5 (2)	C13—C14—H14A	108.5
C9—C8—H8	119.8	C15—C14—H14A	108.5
C7—C8—H8	119.8	C13—C14—H14B	108.5
C10—C9—C8	122.7 (3)	C15—C14—H14B	108.5
C10—C9—H9	118.7	H14A—C14—H14B	107.5
C8—C9—H9	118.7	C14—C15—H15A	109.5
C9—C10—C11	116.1 (2)	C14—C15—H15B	109.5
C9—C10—C13	121.7 (3)	H15A—C15—H15B	109.5
C11—C10—C13	122.2 (3)	C14—C15—H15C	109.5
C10—C11—C12	122.3 (3)	H15A—C15—H15C	109.5
C10—C11—H11	118.8	H15B—C15—H15C	109.5
C12—C11—H11	118.8	C16—N1—C7	121.3 (2)
C7—C12—C11	120.7 (3)	C4—O1—C18	118.6 (2)
C7—C12—H12	119.7	C6—O2—H2	109.5
C11—C12—H12	119.7	C2—O3—C17	118.41 (18)
N1—C16—C1	122.7 (2)		
			
C6—C1—C2—O3	179.7 (2)	C8—C9—C10—C11	−0.2 (4)
C16—C1—C2—O3	1.6 (3)	C8—C9—C10—C13	179.5 (3)
C6—C1—C2—C3	−0.3 (4)	C9—C10—C11—C12	−0.4 (4)
C16—C1—C2—C3	−178.5 (2)	C13—C10—C11—C12	180.0 (3)
O3—C2—C3—C4	−179.5 (2)	C8—C7—C12—C11	0.6 (4)
C1—C2—C3—C4	0.5 (4)	N1—C7—C12—C11	179.0 (2)
C2—C3—C4—O1	179.3 (2)	C10—C11—C12—C7	0.2 (4)
C2—C3—C4—C5	−0.1 (4)	C2—C1—C16—N1	−179.5 (2)
O1—C4—C5—C6	180.0 (2)	C6—C1—C16—N1	2.5 (4)
C3—C4—C5—C6	−0.6 (4)	C9—C10—C13—C14	66.0 (4)
C4—C5—C6—O2	−178.8 (2)	C11—C10—C13—C14	−114.4 (4)
C4—C5—C6—C1	0.8 (4)	C10—C13—C14—C15	178.4 (3)
C2—C1—C6—O2	179.2 (2)	C1—C16—N1—C7	−178.7 (2)
C16—C1—C6—O2	−2.7 (4)	C12—C7—N1—C16	166.3 (2)
C2—C1—C6—C5	−0.3 (4)	C8—C7—N1—C16	−15.5 (4)
C16—C1—C6—C5	177.8 (2)	C5—C4—O1—C18	−174.5 (2)
C12—C7—C8—C9	−1.1 (4)	C3—C4—O1—C18	6.1 (4)
N1—C7—C8—C9	−179.3 (2)	C3—C2—O3—C17	6.2 (4)
C7—C8—C9—C10	0.9 (4)	C1—C2—O3—C17	−173.8 (2)

### Hydrogen-bond geometry (Å, °)

**Table:**

*D*—H···*A*	*D*—H	H···*A*	*D*···*A*	*D*—H···*A*
O2—H2···N1	0.82	1.87	2.602 (2)	148
C18—H18B···Cg2^i^	0.96	2.80	3.764 (3)	178

Symmetry codes: (i) −*x*+1, *y*+1/2, −*z*+3/2.

## References

[bb1] Bernstein, J., Davis, R. E., Shimoni, L. & Chang, N.-L. (1995). *Angew. Chem. Int. Ed. Engl.* **34**, 1555–1573.

[bb2] Calligaris, M., Nardin, G. & Randaccio, L. (1972). *Coord. Chem. Rev.* **7**, 385–403.

[bb3] Cohen, M. D., Schmidt, G. M. J. & Flavian, S. (1964). *J. Chem. Soc.* pp. 2041–2051.

[bb4] Dey, D. K., Dey, S. P., Elmalı, A. & Elerman, Y. (2001). *J. Mol. Struct.* **562**, 177–184.

[bb5] Farrugia, L. J. (1997). *J. Appl. Cryst.* **30**, 565.

[bb6] Farrugia, L. J. (1999). *J. Appl. Cryst.* **32**, 837–838.

[bb7] Hadjoudis, E., Vitterakis, M., Moustakali, I. & Mavridis, I. (1987). *Tetrahedron*, **43**, 1345–1360.

[bb8] Hökelek, T., Kılı˛c, S., Işıklan, M. & Toy, M. (2000). *J. Mol. Struct.* **523**, 61–69.

[bb9] Karadayı, N., Gözüyeşil, S., Güzel, B. & Büyükgüngör, O. (2003). *Acta Cryst.* E**59**, o161–o163.

[bb10] Sheldrick, G. M. (2008). *Acta Cryst.* A**64**, 112–122. 10.1107/S0108767307043930 18156677

[bb11] Stoe & Cie (2002). *X-RED* and *X-AREA* Stoe & Cie, Darmstadt, Germany.

[bb12] Ünver, H., Kabak, M., Zengin, D. M. & Durlu, T. N. (2002). *J. Chem. Crystallogr.* **31**, 203–209.

